# Suppression of Eosinophil Integrins Prevents Remodeling of Airway Smooth Muscle in Asthma

**DOI:** 10.3389/fphys.2016.00680

**Published:** 2017-01-09

**Authors:** Andrius Januskevicius, Reinoud Gosens, Raimundas Sakalauskas, Simona Vaitkiene, Ieva Janulaityte, Andrew J. Halayko, Deimante Hoppenot, Kestutis Malakauskas

**Affiliations:** ^1^Laboratory of Pulmonology, Department of Pulmonology, Lithuanian University of Health Sciences (LSMU)Kaunas, Lithuania; ^2^Department of Molecular Pharmacology, University of GroningenGroningen, Netherlands; ^3^Department of Pulmonology, Lithuanian University of Health Sciences (LSMU)Kaunas, Lithuania; ^4^Department of Physiology and Pathophysiology, University of ManitobaWinnipeg, MB, Canada

**Keywords:** airway smooth muscle, eosinophils, integrins, adhesion, TGF-β_1_, asthma

## Abstract

**Background:** Airway smooth muscle (ASM) remodeling is an important component of the structural changes to airways seen in asthma. Eosinophils are the prominent inflammatory cells in asthma, and there is some evidence that they contribute to ASM remodeling via released mediators and direct contact through integrin–ligand interactions. Eosinophils express several types of outer membrane integrin, which are responsible for cell–cell and cell–extracellular matrix interactions. In our previous study we demonstrated that asthmatic eosinophils show increased adhesion to ASM cells and it may be important factor contributing to ASM remodeling in asthma. According to these findings, in the present study we investigated the effects of suppression of eosinophil integrin on eosinophil-induced ASM remodeling in asthma.

**Materials and Methods:** Individual combined cell cultures of immortalized human ASM cells and eosinophils from peripheral blood of 22 asthmatic patients and 17 healthy controls were prepared. Eosinophil adhesion was evaluated using eosinophil peroxidase activity assay. Genes expression levels in ASM cells and eosinophils were measured using quantitative real-time PCR. ASM cell proliferation was measured using alamarBlue® solution. Eosinophil integrins were blocked by incubating with Arg-Gly-Asp-Ser peptide.

**Results:** Eosinophils from the asthma group showed increased outer membrane α_4_β_1_ and αMβ_2_ integrin expression, increased adhesion to ASM cells, and overexpression of TGF-β_1_ compared with eosinophils from the healthy control group. Blockade of eosinophil RGD-binding integrins by Arg-Gly-Asp-Ser peptide significantly reduced adhesion of eosinophils to ASM cells in both groups. Integrin-blocking decreased the effects of eosinophils on TGF-β_1_, WNT-5a, and extracellular matrix protein gene expression in ASM cells and ASM cell proliferation in both groups. These effects were more pronounced in the asthma group compared with the control group.

**Conclusion:** Suppression of eosinophil-ASM interaction via RGD-binding integrins attenuates eosinophil-induced ASM remodeling in asthma.

**Trial Registration:** ClinicalTrials.gov Identifier: NCT02648074.

## Introduction

Asthma is a heterogeneous disease with imbalanced airway tissue repair associated with airway inflammation that is rich in eosinophils (Scott and Wardlaw, 2006; Blanchard and Rothenberg, 2009). With asthma, structural changes in the airways, called airway remodeling, includes subepithelial fibrosis, increased smooth muscle mass (Holgate, 2000; Halayko et al., 2006), enlargement of glands, neovascularization (Tanaka et al., 2003), and epithelial alterations (Bousquet et al., 2000). An important determinant of airflow limitation in asthma is the degree of airway smooth muscle (ASM) remodeling, which is not a well-studied factor in the pathophysiology of asthma. It includes ASM thickening that is a result from increased ASM cells proliferation and extracellular matrix (ECM) proteins expression (Dekkers et al., 2009). Pathobiological mechanisms for airway remodeling are not fully elucidated, but there appears to be a direct correlation with chronic inflammatory conditions in the airways (Fajt and Wenzel, 2015).

In asthmatic lungs there is increased abundance of growth factors and inflammatory mediators that can directly stimulate the multifunctional behavior of ASM cells (Ngoc et al., 2005). A primary source of cytokines, chemokines and growth factors released in the lungs are inflammatory cells, including eosinophils (Cheung et al., 2008). However, there is not a robust body of evidence that clarifies the details of how inflammatory cells contribute to ASM remodeling. Eosinophil infiltration in the bronchial mucosa is controled by various cytokines and chemokines, such as interleukin (IL) -5, -13 the eotaxins, RANTES, and involves signaling via Rho-kinase (Chiappara et al., 2001; Taki et al., 2007; Murdoch and Lloyd, 2010; Possa et al., 2012). Activated by IL-5 family cytokines, eosinophils are significant source of transforming growth factor-β1 (TGF-β1) which can induce hypertrophic and hyperplastic ASM cell growth (Chen and Khalil, 2006; Xiao et al., 2012). TGF-β1 is a key mediator involved in tissue remodeling in the asthmatic lung and eosinophils are the main cell type that produce this profibrotic cytokine in asthma. Many studies have reported increased levels of TGF-β1 in asthmatic patients, but the main mechanisms behind this increase remain unclear. TGF-β1 has the ability to induce a transformational change in normal cells that effects a number of cellular biological processes as epithelial changes, subepithelial fibrosis and microvascular changes leading to airway remodeling (Halwani et al., 2011). Moreover TGF-β1 can promote ECM protein biosynthesis via canonical Smad-signaling (Sethi et al., 2011) as well as non-canonical WNT signaling (Kumawat et al., 2013). Non-canonical Wnt signaling can contribute to ASM remodeling via ECM protein production (Kumawat et al., 2013). Altered structural function of type-1 collagen and the amount of fibronectin can act not only as a proliferative signal for structural cells but can also play a part in increasing ASM mass in asthma (Johnson et al., 2004).

There is evidence that eosinophils contribute to ASM remodeling through direct contact via integrin-ligand interaction (Hughes et al., 2000). Integrins are dimeric transmembrane receptors that are the link for cell–cell and cell–ECM interactions. Cell adhesion through integrins can trigger a signal transduction that controls cells growth, division, survival, cellular differentiation, and apoptosis (Kim et al., 2011; Carbonell et al., 2013). Seven integrin heterodimers expressed by eosinophils (α_4_β_1_, α_6_β_1_, α_L_β_2_, α_M_β_2_, α_X_β_2_, α_D_β_2_, and α_4_β_7_) mediate diverse functions, including eosinophil rolling, stable adhesion, migration, respiratory bursts, degranulation, and viability after interaction with ligands including adhesion molecules, laminin, fibrinogen/fibrin, vitronectin, and periostin on other cells or in the ECM (Barthel et al., 2008; Johansson and Mosher, 2013; Johansson et al., 2013). ASM cells express VCAM-1 and ICAM-1 adhesion molecules, which can act as ligands for eosinophils integrins α4β1 and αMβ2 and previous study revealed that the adhesion ability of eosinophils in asthma patients is significantly greater than the adhesion ability of eosinophils from healthy people (Januskevicius et al., 2016). On this basis, the potential that blocking eosinophil surface integrins as a means to suppress the impact of inflammation on airway structural cells and airway remodeling, has emerged as a potential therapeutic approach for asthma (Dekkers et al., 2010).

We previously demonstrated that asthmatic eosinophils significantly enhance ASM cells proliferation comparing with healthy eosinophils and it may be related with increased eosinophils adhesion and their effect on TGF-β_1_, WNT-5a and ECM proteins production (Januskevicius et al., 2016). These findings led us to investigate possible ways how to prevent eosinophils effects on ASM remodeling in asthma. There are few suppressing agents targeting eosinophil activity that can be used *in vitro* to control eosinophil-induced ASM remodeling. We tested the hypothesis that using the small tetrapeptide Arg-Gly-Asp-Ser (RGDS) to modulate integrin-associated eosinophi interactions with ASM cells can blunt the pro-remodeling function of ASM cells *in vitro*. RGDS peptide is a non-selective integrin receptor antagonists which mainly works through α4β_1_ integrin or αMβ_2_ integrin (Ahmadzai et al., 2015). These integrins can recognize Ar-Gly-Asp amino acids motifs on VCAM-1 or ECM proteins (Ruoslahti, 1996; Takagi, 2004). Suppression of these integrins by RGDS peptide can block the natural function of eosinophils to attach to airway structural cells or ECM proteins after migration from peripheral blood. We investigated the increased proliferation-promoting feature of eosinophils from the asthma group with the aim of controlling proliferation through blocking eosinophil attachment to the surface of ASM cells.

## Materials and methods

### Subjects

Thirty-nine nonsmoking adults (men and women) were included into the study: 22 patients with persistent mild to moderate asthma defined according the GINA criteria and 17 healthy subjects who comprised the control group. The patients were recruited from the Department of Pulmonology and Immunology, Hospital of the Lithuanian University of Health Sciences, Kaunas. The study protocol was approved by the Regional Biomedical Research Ethics Committee of the Lithuanian University of Health Sciences (BE-2-13). Each participant was informed about ongoing investigation and gave his/her written consent.

Patients with asthma had a clinical history of the disease for ≥1 year, current symptoms, airway hyperresponsiveness and positive skin prick test (≥3 mm) in response to house dust mites *Dermatophagoides pteronyssinus* (*D. pteronyssinus*) or *Dermatophagoides farinae* (*D. farinae*). All patients were not using inhaled, nasal or oral steroids at least 1 month before visits, short-acting β_2_ agonists at least 12 h and long-acting β_2_ agonists at least 48 h prior the lung function test, antihistamines and antileukotrienes—7 days before skin prick test and prior the lung function test. None of the patients had a history of smoking. Baseline forced expiratory volume in 1 s (FEV1) was more than 70% of the predicted value in all patients. All healthy subjects were nonsmokers, without symptoms of rhinitis or asthma, with normal findings of spirometry and methacholine challenge, and all showed negative results of skin prick test. Demographic and clinical data are presented in Table 1.

**Table 1:** **Demographic, clinical and laboratory data of study subjects**.

**Characteristics**	**Asthma patients *n* = 22**	**Healthy subjects *n* = 17**
Age (years)	32.0 ± 1.9	27.7 ± 1.2
Gender (M/F), n	16/6	10/7
FEV_1_ (L)	3.71 ± 0.17	3.93 ± 0.19
FEV_1_(% of predicted)	104.6 ± 2.55	101.1 ± 1.9
Wheal diameter induced by allergen (mm)	4.53 ± 1.4	0
PD_20_ (mg)	0.05^#^	NR
Blood eosinophil count (× 10^9^/L)	0.32 ± 0.04^*^	0.14 ± 0.02
Sputum eosinophil count (× 10^9^/L)	0.10 ± 0.02^*^	0.03 ± 0.004

*F, female; M, male; FEV_1_, forced expiratory volume in 1 s; PD_20_, the provocation dose of methacholine causing a 20% decrease in FEV_1_; NR, not responded. Data presented as Mean ± standard error of the mean*.

* *p < 0.01 comparing with healthy subjects peripheral eosinophils count*.

# *geometric mean*.

### Lung function testing

Pulmonary function was tested using a pneumotachometric spirometer “CustovitM” (Custo Med, Germany). Baseline forced expiratory volume in 1 s (FEV1), forced vital capacity (FVC), and FEV1/FVC ratio were recorded as the highest of three reproducible measurements. The results were compared with the predicted values matched for age, body height, and sex according to the standard methodology.

### Measurement of airway responsiveness to methacholine

Airway responsiveness was assessed as changes in airway function after challenge with inhaled methacholine using a reservoir method. Methacholine was nebulized into a 10-L reservoir with a pressure nebulizer (Pari Provocation I; Pari, Stanberg, Germany). Aerolized methacholine was inhaled through a one-way valve at 5-min intervals starting with 15-μg methacholine dose and doubling it until a 20% decrease in FEV1 from the baseline or the total cumulative dose of 3.87 mg was achieved. The bronchoconstricting effect of each dose of methacholine was expressed as a percentage of decrease in FEV1 from the baseline value. The provocative dose of methacholine causing a ≥20% fall in FEV1 (PD20) was calculated from the log dose-response curve by linear interpolation of two adjacent data points.

### Skin prick testing

All patients were screened for allergy by the skin prick test using standardized allergen extracts (Stallergenes S.A., France) for the following allergens: D. pteronyssinus, D. farinae, cat and dog dandruff, 5 mixed grass pollen, birch pollen, mugwort, Alternaria, Aspergillus, and Cladosporium. Temoin was used for a negative control, and histamine hydrochloride (10 mg/mL) was used for a positive control. Skin testing was read 15 min after application. The results of skin prick test were considered positive if the mean wheal diameter was ≥3 mm.

### Sputum induction, processing, and cells analysis

After baseline FEV1 and FVC measurements, subjects inhaled 10 mL of sterile hypertonic saline solution (3, 4, or 5% NaCl, Ivex Pharmaceuticals, USA) at room temperature. Nebulised solution was given for three periods of 5 min at most by an ultrasonic nebuliser ULTRA-NEB™ (DeVilbiss, USA). The subjects were instructed to cough sputum into containers. If any symptoms occurred, nebulisation was discontinued. It was stopped after expectoration of an adequate amount of sputum. In order to detect a possible decrease in FEV1, spirometry was performed after each inhalation. Sputum was poured into a Petri dish and separated from saliva. A 4-fold volume of freshly prepared 0.1% dithiothreitol (DTT; Sigma-Aldrich) was added. The mixture was vortexed and placed on ice and rocked on an orbital shaker OS-10 (BIOSAN, Latvia) for 15 min. Next, an equal volume of phosphate-buffered saline solution (PBS; Sigma-Aldrich) was added to DTT. The cell pellet was separated using a 40-μm cell stainer (Becton Dickinson, USA). The mixture was centrifuged for 10 min at 4°C; the supernatant was aspirated and stored at −70°C for later analysis. The total cell counts, percentage of epithelial cells, and cell viability were investigated using a Neubauer hemocytometer (Heinz-Herenz, Germany) under a microscope (BX43, OLYMPUS, USA) by employing the Trypan blue exclusion method. The cytospin samples of induced sputum were prepared using a cytofuge instrument (Shandon Southern Instruments, USA). Supernatant with and without DTT was stored in 2 mL microtubes at −70°C for further researches. The prepared sputum cytospins were stained by the May-Grünwald-Giemsa method for differential cell counts. Cell differentiation was determined by counting approximately 400 cells in random fields of view under a light microscope, excluding squamous epithelial cells. The cells were identified by standard morphological criteria, nuclear morphology, and cytoplasmic granulation.

### Peripheral blood collection and isolation of eosinophils

Peripheral blood samples for eosinophil isolation were collected into sterile Vacutainer tubes with EDTA. Polymorphonuclear leukocytes (PMN) were isolated by high-density gradient centrifugation. The whole blood was layered on Ficoll-Paque PLUS (GE Healthcare, Finland) and centrifuged at 1000 g force for 30 min at room temperature, and then PMN were separated by hypotonic lysis of erythrocytes. Eosinophils were separated using a magnetic eosinophil isolation kit (Miltenyi Biotec, USA) according to the procedure previously described (Januskevicius et al., 2016). Manufacturer confirms that eosinophils separation kit do not influence eosinophils viability and separation efficiency are more than 97%. Eosinophils were counted using an ADAM automatic cell counter (Witec AG, Switzerland)—eosinophils viability must to be found at least 98%. To check eosinophil purity after magnetic separation, a diluted eosinophil suspension was fixed with methanol on a glass plate, stained with May–Grunwald–Giemsa dyes, and inspected by light microscopy to determine whether there was any other cell in the suspension.

### ASM cell culture

Healthy human ASM cells, immortalized by stable expression of hTERT, as described previously (Gosens et al., 2006), were used for experiments. For all experiments, the same hTERT-ASM cell line was used at Passage 5 for all 39 investigating subjects, thus avoiding changes in ASM activity and viability that could result from repeated thawing and passage. Cells were cultivated in 75 cm^2^ Falcon culture flasks under standard culture conditions of 5% CO_2_ in air at 37°C with medium renewal every 2–3 days. For all experiments, passaged cells were grown on plastic dishes in Dulbecco's modified eagle medium (DMEM) (GIBCO®; Life Technologies, Paisley, UK) supplemented with streptomycin/penicillin (2% v/v; GIBCO®; Life Technologies), amphotericin B (1% v/v; GIBCO®; Life Technologies), and fetal bovine serum (10% v/v; GIBCO®; Life Technologies). After reaching sufficient confluency, cells were passaged by trypsinization. Cells were serum deprived in DMEM supplemented with antibiotics and insulin, transferrin, and selenium reagent (GIBCO®; Life Technologies) before each experiment to stop cell proliferation and avoid possible errors in proliferation and gene expression measurements due to the effects of mediators of bovine serum in growth medium.

### Co-culture of ASM cells and eosinophils

Whole eosinophils were separated into 3 groups depended by peptides were incubated with: control eosinophils—without incubation with peptides; eosinophils incubated with integrins blocking peptide RGDS; eosinophils incubated with integrins non-blocking peptide (negative control) GRADSP. Respectively amount of eosinophils suspension in serum-free growth medium was taken and peptide solution was added to the final concentration of 0.125 mg/ml. Eosinophils with peptides were incubated for 1 h at 37°C. After incubation eosinophils were centrifuged, growth medium was removed and fresh serum-free growth medium was added.

Cell cultivation dishes with approximately 2 × 10^5^ ASM cells were prepared and co-cultures was made by adding eosinophil suspension with 0.5 × 10^5^ viable eosinophils to the ASM cells.

To observe and visualize cell growth, an inverted microscope (CETI Inverso TC100; Medline Scientific, Oxford, UK) was used which had a 10 × /22 mm wide-field eyepiece, a phase objective of 10 × /0.25, and an installed CETI Si-5C camera (Si-Cap software, ver. 2.1, 2012).

### Eosinophil adhesion assay

Airway smooth muscle (ASM) cells were seeded in six-well plates (2 × 10^4^ per well) and grown in DMEM//10% FBS for 3 days. Medium was removed and wells were washed twice with warm PBS. In serum-free DMEM (supplemented with 1% insulin-transferrin-selenium reagent) cells were incubated for 1 day, after which a co-culture with eosinophils was prepared.

Eosinophil adhesion was measured for up to 240 min thereafter, removing non-adherent cells by aspiration, then washing cultures twice with warm PBS. Adherent eosinophil abundance in the cultures was determined by measuring residual eosinophil peroxidase (EPO) activity as previously described (White et al., 1991). Because intercellular EPO levels may be intrinsically lower in eosinophils from asthmatic individuals owing to degranulation (Krug et al., 1999), increased EPO protein levels detected by eosinophil adhesion assay in the co-culture models can be assigned as a higher number of eosinophils stably adhering to the ASM cell surface.

To assay EPO activity, 462 μL DMEM without phenol red and 462 μL EPO substrate (1 mM H_2_O_2_, 1 mM *o*-phenylenediamine, 0.1% Triton X-100 in Tris buffer; pH 8.0) were added to each well. After 30 min at 37°C, 231 μL of 4 M H_2_SO_4_ was added to each well to stop the reaction and absorbance was measured at 490 nm using a microplate reader. Results are expressed relative to substrate oxidation levels in control cells (ASM cells without incubation with eosinophils). Light absorbance in control cell wells is presented as 100% and eosinophil adhesion is expressed as the difference from the control value.

### Alamarblue® proliferation assay

Cells for proliferation measurements were grown in six-well cluster plates in conditions described previously. Healthy ASM cells were co-cultured with their respective eosinophil group isolated from asthma patients and healthy individuals for 72 h. Following co-culture, cells were washed once with warm PBS, incubated for 4 min with warm PBS supplemented with 1 mM EDTA to detach eosinophils from ASM cells, and washed again with warm PBS to remove any remaining EDTA. Proliferation was evaluated by incubating wells with Hank's balanced salt solution containing alamarBlue® (10% v/v; Invitrogen™; Life Technologies, Paisley, UK). Conversion of alamarBlue® to the reduced form was dependent on the metabolic activity of ASM cells and was assayed by dual-wavelength spectrophotometry at wavelengths of 570 nm and 600 nm. As indicated by the manufacturer, the degree of alamarBlue® conversion is proportional to the number of viable cells. Data are expressed as the percentage increase or decrease in alamarBlue® conversion by ASM cells compared with control cells (ASM cells cultured without eosinophils), which did not proliferate during the co-culturing period because of serum-free growth medium.

### Detection of protein levels in co-culture growth medium

TGF-β_1_ and ECP protein levels in co-culture growth medium were measured using ELISA according to the manufacturer's instructions. Twenty-four hours before experiments, growth medium with fetal bovine serum was changed to fresh serum-free medium at the same volume for all wells. After a 24-h co-culture, growth medium was collected in new 10 mL tubes, centrifuged at 400 × *g* for 10 min at 25°C to separate non-attached cells, and 2 mL cryogenic tubes containing 1 mL supernatant were frozen at −80°C for protein level analysis. The results are expressed as protein level per 1 mL growth medium. LDL for TGF-β_1_ is 23.44 pg/mL, for ECP–1.9 pg/ml.

### RNA isolation and real-time PCR

Total RNA was extracted using a miRNeasy mini kit (Qiagen, Valencia, CA) according to the manufacturer's instructions. Real-time PCR was performed using a Power SYBR® Green RNA-to-C_T_™ 1-Step kit (Applied Biosystems, Foster City, CA) in a 7500 Fast Real-Time PCR system as follows: reverse transcription at 48°C for 30 min; activation of AmpliTaq Gold® DNA polymerase, UP (Ultra-Pure) at 95°C for 10 min; 40 cycles of denaturation at 95°C for 15 s; and annealing and extension at 60°C for 1 min. Real-time PCR data were analyzed using the comparative cycle threshold method (the amount of target gene was normalized to the endogenous reference gene 18S ribosomal RNA). A difference of 1 C_t_ (ΔC_t_ = 1, where C_t_ is the amplification cycle number) after normalization to the reference gene indicates a 2-fold higher expression level of the investigated gene. Relative differences were determined by normalization of test sample ΔC_q_ values to control sample ΔC_q_ values with the equation ΔC_q_(control)−ΔC_q_(test), and the differences in fold were expressed by 2^ΔΔCt^ value. Primers used to analyze gene expression are shown in Table 2.

**Table 2:** **Sequences of primers used for gene expression analysis**.

**Primer**	**Forward 5′−3′**	**Reverse 5′−3′**
TGF-β_1_	GTACCTGAACCCGTGTTGCT	GAACCCGTTGATGTCCACTT
Wnt-5a	GGGTGGGAACCAAGAAAAAT	TGGAACCTACCCATCCCATA
fibronectin	TCGAGGAGGAAATTCCAATG	ACACAGTGCACCTCATCAT
collagen type Iα1	AGCCAGCAGATCGAGAACAT	TCTTGTCCTTGGGGTTCTTG
α_4_	GCTTCTCAGATCTGCTCGTG	GTCACTTCCAACGAGGTTTG
α_M_	CAGACAGGAAGTAGCAGCTCCT	CTGGTCATGTTGATGAAGGTGCT
β_1_	GTGTGGCCCAAGACAGTTCT	GGTTACCCCACCCTCTGACT
β_2_	CAACGTATGCGAGTGCCATTC	TCACGGGGTTGTTCGACAG
18S	CGCCGCTAGAGGTGAAATTC	TTGGCAAATGCTTTCGCTC

### Statistical analysis

Statistical analysis was performed with GraphPad Prism 6 for Windows (ver. 6.05, 2014; GraphPad Software Inc., San Diego, CA). Normally distributed data are mean and standard error of the mean (SEM). Significant differences between two independent groups were determined using the Mann–Whitney *U*-test. The Wilcoxon matched-pairs signed rank test was used for dependent groups. A *p* < 0.05 was considered statistically significant.

## Results

### Characteristics of the studied participants

The main characteristics of the study participants are shown in Table 1. A total of 39 non-smoking adults participated in the study (22 steroid-free patients with intermittent or mild-to-moderate persistent asthma, and 17 healthy participants who constituted the control group). There were no significant differences in age, gender balance or FEV_1_ existed between groups, however, the asthma group was unique in exhibiting airway hyperresponsiveness (PD20) as well as skin prick positivity. Notably, all asthma patients had significantly higher numbers of peripheral blood eosinophils, and increased eosinophil number in induced sputum (Table 1).

### Eosinophil integrin expression and adhesion to ASM cells

**Figure 1:**
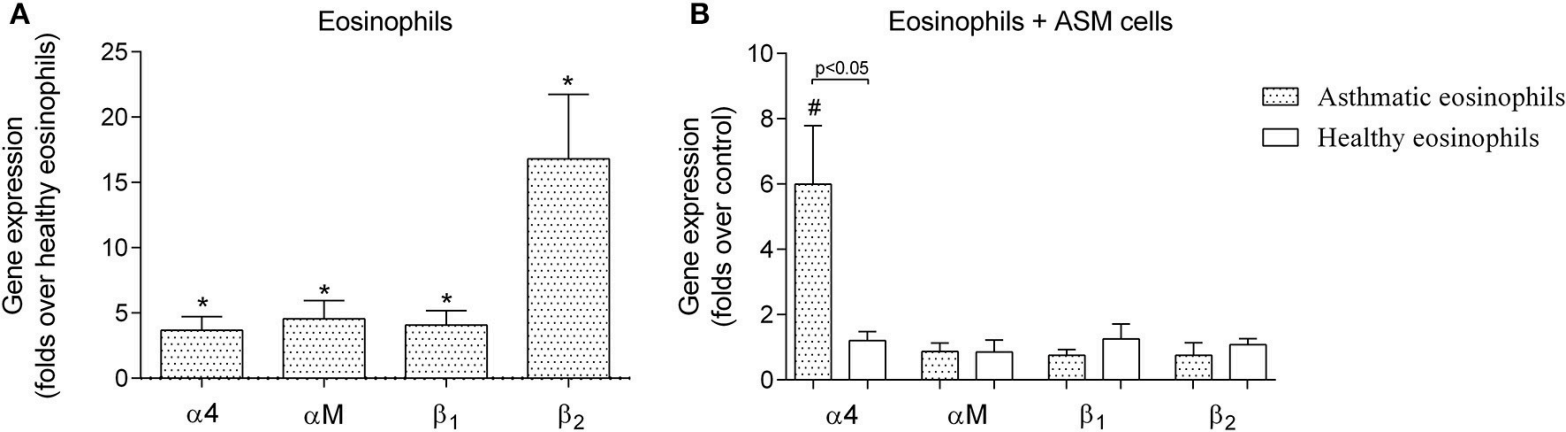
**Gene expression of eosinophil integrin. (A)** Integrin gene expression differences between eosinophils isolated from asthmatic patients compared with eosinophils from the healthy control group. **(B)** Influence of a 24-h incubation with airway smooth muscle (ASM) cells on gene expression of eosinophil integrin. Results are mean ± SEM. Asthma group *n* = 22; control group *n* = 17. ^*^p < 0.05 compared with healthy eosinophils; ^#^*p* < 0.05 compared with control group (eosinophils that had not been incubated with ASM cells).

We observed that asthmatic eosinophils had 3.7 ± 1.0-fold of α4, 4.6 ± 1.4-fold of αM, 4.1 ± 1.1-fold of β_1_ and 16.8 ± 4.9-fold of β_2_ more mRNA, compared to eosinophils from healthy individuals (Figure 1A). We also determined the effects of eosinophil adhesion to cultured human ASM cells on the abundance of α4, αM, β_1_, and β_2_ mRNA (Figure 1B). We collected adherent asthmatic and healthy eosinophils after 24 h of co-culture with ASM cells, and determined that asthmatic eosinophils exhibited a 6.0 ± 1.8-fold increase in α4 mRNA compared to eosinophil that were not incubated with ASM cells (*p* < 0.05). In striking contrast, eosinophils from healthy donors exhibited no change in α4, β1, and β2 mRNA after 24 co-culture with human ASM cells (Figure 1B). We measured no change in other integrins subunits mRNA abundance after co-culture with ASM cells in eosinophils from either healthy or asthmatic donors.

**Figure 2:**
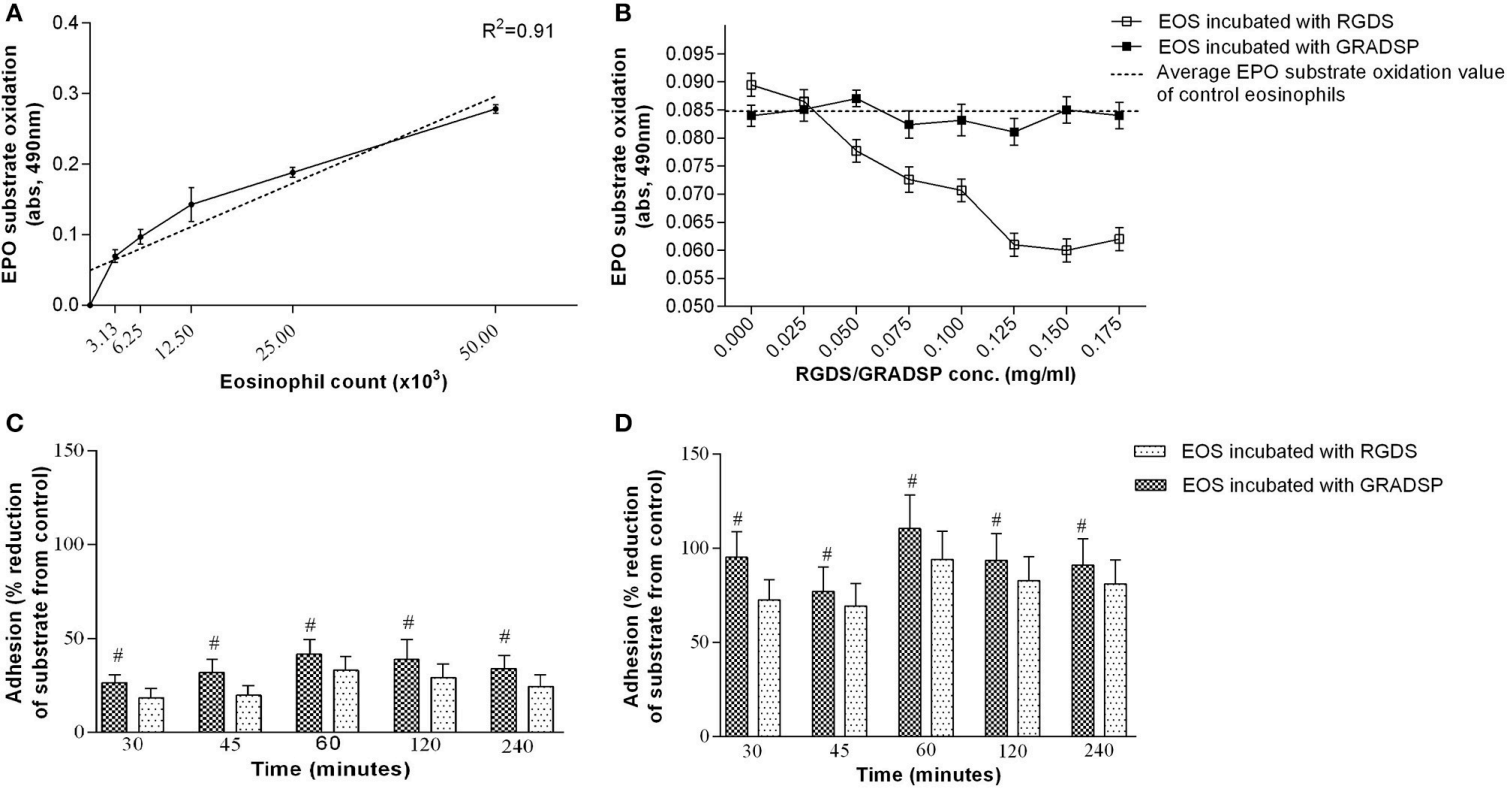
**Measurement of eosinophil adhesion. (A)** Calibration curve of eosinophil peroxidase (EPO) substrate oxidation dependency by eosinophil count. **(B)** Eosinophil integrin-suppression efficiency at different Arg-Gly-Asp-Ser (RGDS)/Gly-Arg-Ala-Asp-Ser-Pro (GRADSP) concentrations. **(C)** Effects of blocking integrin adhesion to airway smooth muscle (ASM) cells in the control group (healthy participants) at various periods of incubation. **(D)** Effects of blocking integrin adhesion to ASM cells in the asthma group at various periods of incubation. Results are mean ± SEM. Asthma group *n* = 22; control group *n* = 17. Control: ASM cells that had not been co-cultured with eosinophils; ^#^*p* < 0.05 compared with eosinophils that had not been subject to integrin-blocking. Integrin-blocking: 1-h incubation with RGDS/GRADSP in a 0.125 mg/mL final concentration.

From the calibration curve, eosinophil number significantly correlated with light absorbance value (Figure 2A). One hour was the optimal period of incubation with RGDS peptide (0.125 mg/mL) for sufficient formation of the electrostatic junction between the eosinophil integrin active center and the amino acid peptides. Moreover, in pre-experiments, we determined the most effective RGDS concentration that would have a significant effect on suppressing eosinophil integrin and determined that negative control GRADSP had no integrins blocking properties as EPO substrate oxidation by non-treated eosinophils and eosinophils incubated with GRADSP was equal (Figure 2B).

For all eosinophil adhesion measurements, the same eosinophil preparation protocol was used. This was a 1-h incubation with 0.125 mg/mL RGDS peptide or its negative control, GRADSP.

Eosinophils isolated from the asthma group showed significantly increased adhesion to ASM cells at all time points, with an average value of 58.83 ± 4.50%. Integrin-blocking peptide had a significant effect on reducing eosinophil adhesion in both the control and asthma groups, with similar percentage values. In the control group, adhesion decreased by an average of 9.54 ± 0.71% (Figure 2C), while blocking of eosinophil integrin in the asthma group reduced adhesion by an average of 13.53 ± 2.70% (Figure 2D).

### TGF-β_1_ production

**Figure 3:**
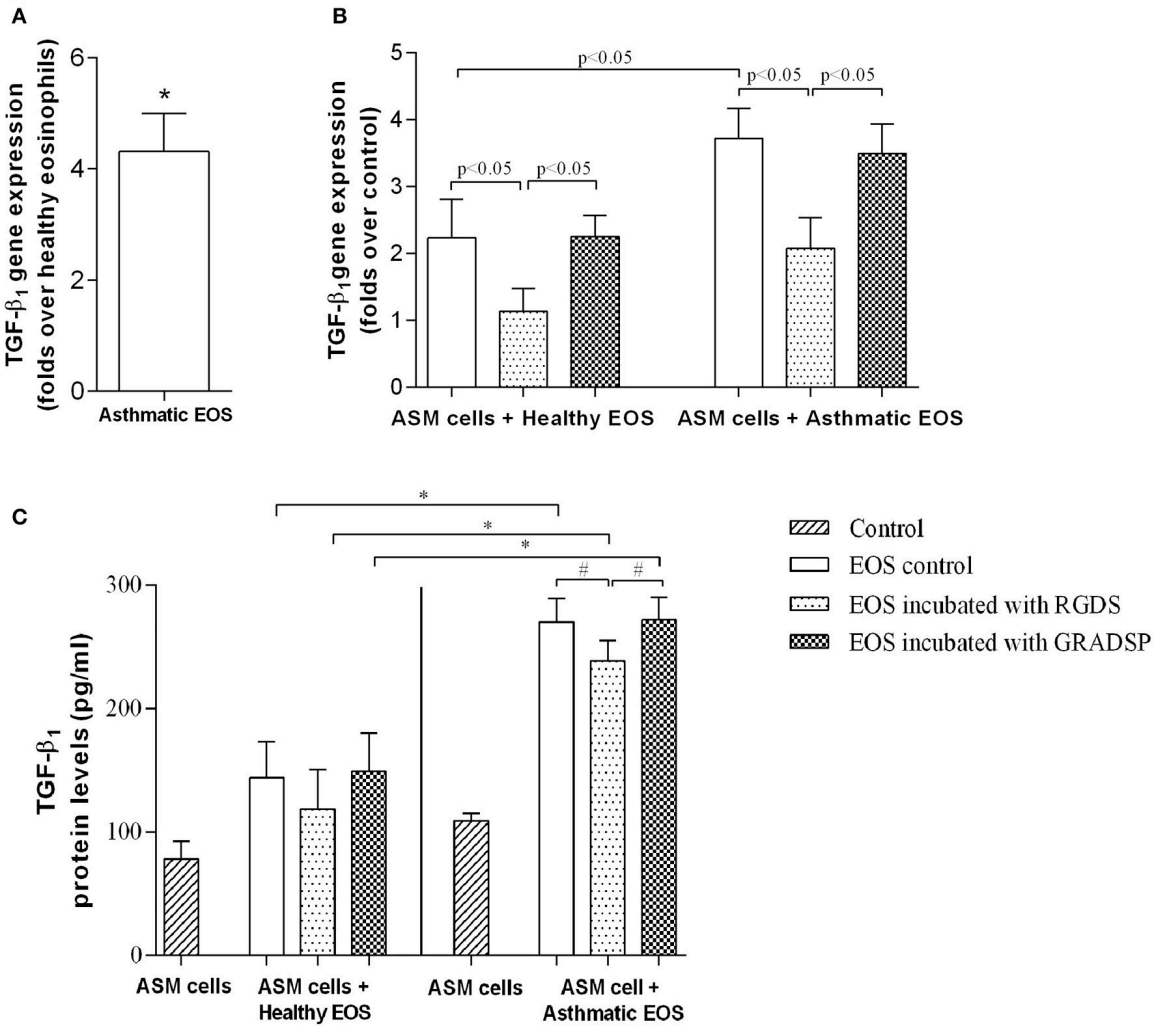
**Production of transforming growth factor-β_1_ (TGF-β_1_). (A)** TGF-β_1_ gene expression differences between eosinophils isolated from asthmatic patients compared with eosinophils from the healthy control group. **(B)** Changes in TGF-β_1_ gene expression in ASM cells after exposure to either eosinophils that had or had not been subject to integrin-blocking. **(C)** Protein levels of free TGF-β_1_ in growth medium of eosinophil and ASM cell co-cultures. Results are mean ± SEM. Asthma group *n* = 22; control group *n* = 17. ^#^*p* < 0.05 compared with eosinophils that had not been subject to integrin-blocking; ^*^p < 0.05 compared with eosinophils from the control group. Control, ASM cells that had not been co-cultured with eosinophils; EOS control, non-treated eosinophils; integrin-blocking, 1-h incubation with Arg-Gly-Asp-Ser (RGDS)/Gly-Arg-Ala-Asp-Ser-Pro (GRADSP) in a 0.125 mg/mL final concentration.

We investigated differences in eosinophil TGF-β_1_ gene expression between the asthma group and the control group. We found that the gene was expressed to a greater extent in the asthma group (4.32 ± 0.68–fold) compared with the control group (Figure 3A). Eosinophils from both groups were observed to induce TGF-β_1_ gene expression in ASM cells, but the effect in the asthma group was significantly higher compared with the control group (asthma group gene overexpression: 3.50 ± 0.44–fold vs. control group gene overexpression: 2.26 ± 0.32–fold; *p* < 0.05) (Figure 3B).

Considering that direct contact with ASM cells is necessary to induce the effects of eosinophils, we investigated changes in TGF-β_1_ gene expression after blocking eosinophil integrins. Incubation with RGDS reduced the influence of eosinophils from the control group from 2.26 ± 0.32–fold to 1.14 ± 0.34–fold, and from 3.50 ± 0.44–fold to 2.08 ± 0.46–fold for the asthma group (*p* < 0.05) compared with eosinophils incubated with GRADSP, the negative control (Figure 3B).

Changes in gene expression had an influence on circulating TGF-β_1_ protein levels in growth medium of co-cultures. With ELISA of co-culture supernatant, we found that the highest protein level was in medium from co-cultures of ASM cells and eosinophils that were not subject to integrin-blocking from the asthma group (270.9 ± 18.5 pg/mL). For the TGF-β_1_ level from ASM cells cultivated alone under the same conditions, there was an average difference of 162.7 pg per mL in the asthma group compared with the control group. In the control group, TGF-β_1_ levels were lower because there was less expression of this growth factor gene in eosinophils and ASM cells after exposure (the concentration in ASM cells cultivated alone was 78.1 ± 14.3 pg/mL, while in co-cultures of control eosinophils and ASM cells, the protein level increased to 143.9 ± 29.1 pg/mL). Blocking eosinophil integrins with RGDS peptide significantly reduced the TGF-β_1_ level in growth medium of co-cultures of eosinophils from the asthma group and ASM cells (reduced by 31 pg. per mL; *p* < 0.05). In the control group, a reduction was also seen, but because of too much variation in data, the *p*-value was over the significance limit (Figure 3C).

### Increase in TGF-β1 production was not associated with the level of eosinophil cationic protein (ECP)

As studies have reported that ECP stimulated TGF-β_1_ production in lung fibroblasts (Zagai et al., 2007), we tested the hypothesis that ECP released from eosinophils can induce overexpression of TGF-β_1_ in ASM cells. To this end, we collected serum-free growth medium from co-cultures of eosinophils and ASM cells and used ELISA to estimate ECP protein levels.

**Figure 4:**
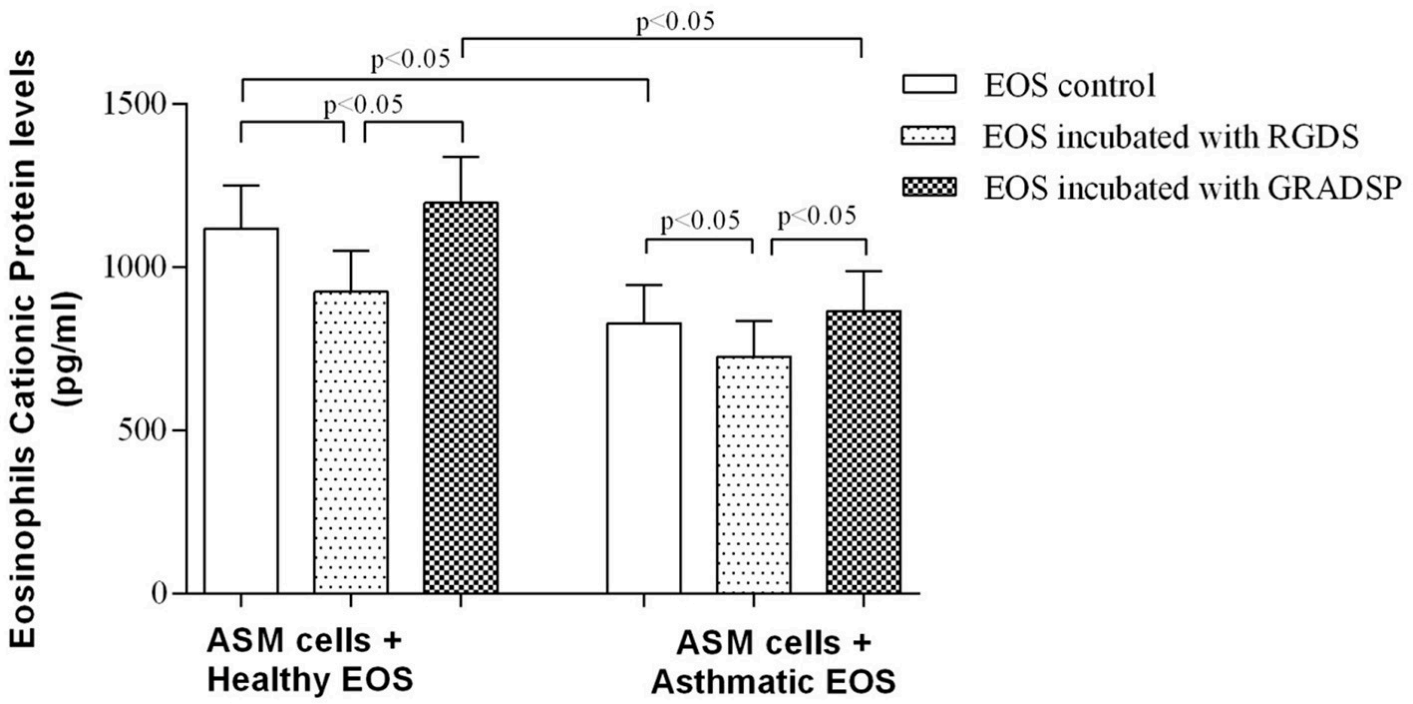
**Eosinophil cationic protein concentration in co-culture growth medium**. Results are mean ± SEM. Asthma group *n* = 22; control group *n* = 17. Negative control, ASM cells that had not been subject to co-culture with eosinophils; EOS control, non-treated eosinophils; integrin-blocking, 1-h incubation with RGDS/GRADSP in a 0.125 mg/mL final concentration.

The highest ECP protein level was observed in medium from co-cultures of control eosinophils without integrin blocking and ASM cells (1118.0 ± 132.4 pg/mL in eosinophils without contact to exogenous proteins, and 1196.0 ± 140.7 in eosinophil incubated with integrin non-binding peptide GRADSP). ECP protein levels were significantly lower in medium containing eosinophils from the asthma group (827.7 ± 118.3 pg per 1 mL in co-cultures with control eosinophils, and 865.2 ± 123.5 pg per 1 mL of growth medium in co-cultures with ASM cells and eosinophils incubated with the negative control GRADSP). Integrin-blocking peptide had a similar effect on reducing ECP production in both the asthma and control groups. Blocking eosinophil integrin in the control group showed a decrease in ECP level of 271.6 pg per 1 mL, whereas the asthma group showed a decrease of 139.6 pg per mL compared with eosinophils incubated with the negative control GRADSP (Figure 4).

### Eosinophils stimulated WNT-5a and ECM protein production

**Figure 5:**
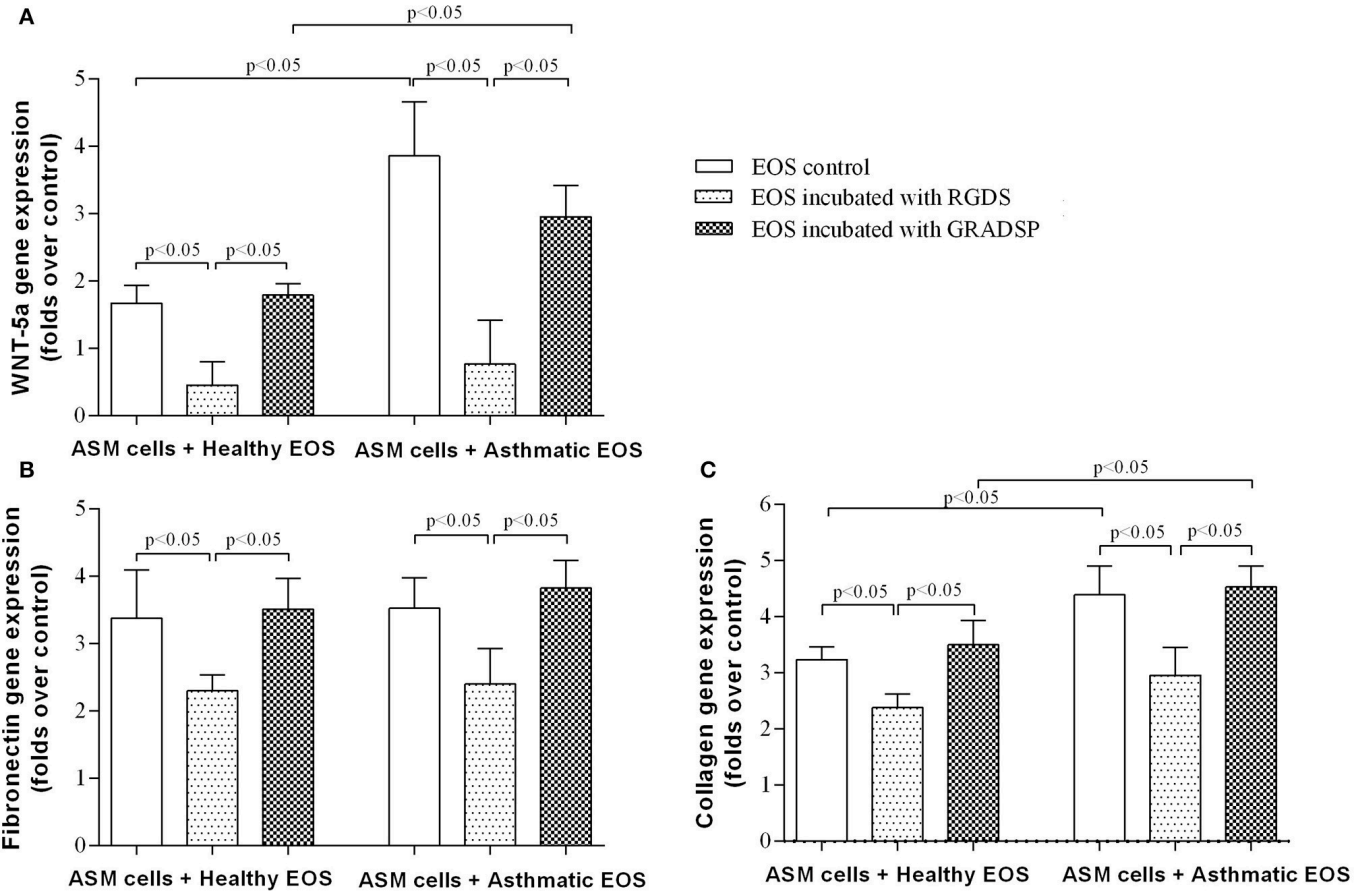
**Effect of eosinophil integrin-blocking on *WNT-5a* and extracellular matrix protein production in airway smooth muscle (ASM) cells. (A)** Influence of eosinophil integrin suppression on *WNT-5a* gene expression in ASM cells. **(B)** Influence of eosinophil integrin suppression on fibronectin gene expression in ASM cells. **(C)** Influence of eosinophil integrin suppression on collagen gene expression in ASM cells. Results are mean ± SEM. Asthma group *n* = 22; control group *n* = 17. Negative control, ASM cells that had not been subject to co-culture with eosinophils; EOS control, non-treated eosinophils: 1-h incubation with Arg-Gly-Asp-Ser (RGDS)/Gly-Arg-Ala-Asp-Ser-Pro (GRADSP) in a 0.125 mg/mL final concentration. Statistical significance, *p* < 0.05.

We found that exposure to eosinophils from both the asthma and control groups increased *WNT-5a* gene expression in ASM cells, but the asthma group showed a significantly higher effect (asthma group increase in gene expression of the ligand: 2.95 ± 0.46–fold vs. control group increase: 1.79 ± 0.16–fold; *p* < 0.05). Reducing the ability of eosinophils to adhere to the surface of ASM cells or ECM protein by integrin-blocking using the RGDS peptide had a significant inhibitory effect on *WNT-5a* gene expression. Decreased TGF-β_1_ levels in co-cultures of ASM cells and asthma or control eosinophils using integrin-blocking reduced *WNT-5a* gene expression, and significant increases in expression were not observed (Figure 5A).

We found that eosinophils from the asthma and control groups showed an increase in fibronectin gene expression in ASM cells by 3.51 ± 0.46–fold and 3.82 ± 0.41–fold, respectively, without any significant differences between the groups (Figure 5B). Eosinophils from the asthma group had a significantly greater effect on type-1 collagen gene expression in ASM cells compared with eosinophils from the control group (eosinophils from the control group showed an increase in gene expression of 3.47 ± 0.37–fold vs. the asthma group increase of 4.67 ± 0.53–fold; *p* < 0.05) (Figure 5C).

Eosinophil integrin-blocking had similar significant effects in both groups. Fibronectin gene expression showed a reduction in the asthma group to 2.50 ± 0.57–fold compared with the control group which was 2.30 ± 0.24–fold. Importantly, suppression of eosinophil integrin reduced the increased effect of eosinophils on collagen gene expression (eosinophils from the control group with integrin-blocking showed an increase in collagen gene expression by 2.46 ± 0.31–fold vs. 2.81 ± 0.53–fold for the asthma group) (Figures 5A,B).

### Eosinophil-induced ASM cell proliferation can be controlled by eosinophil integrin-blocking

Eosinophils isolated from peripheral blood of asthma patients had a greater effect on ASM cell proliferation compared with eosinophils from healthy control participants because of increased adhesion ability. After eosinophils from the asthma group were incubated for 72 h, we found that ASM cells showed increased proliferation of 22.41 ± 3.78%, whereas eosinophils from the control group only increased by 6.90 ± 1.53%.

**Figure 6:**
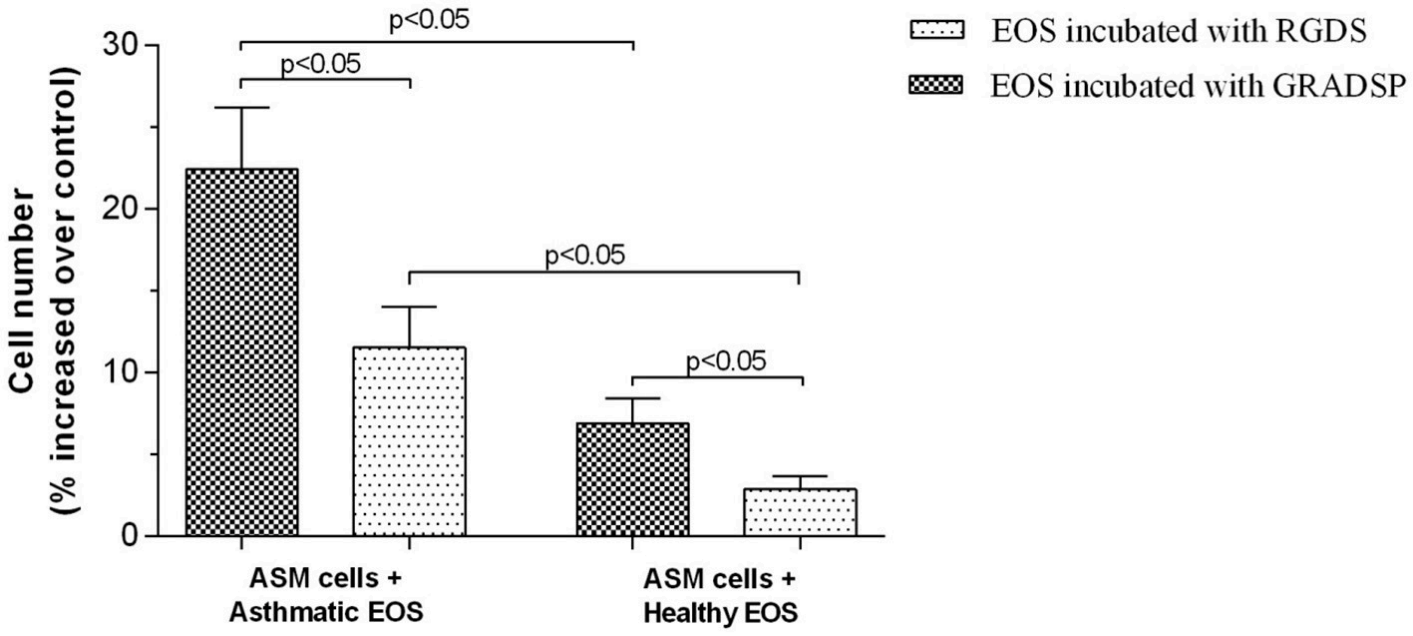
**Airway smooth muscle (ASM) cell proliferation after incubation with eosinophils**. Results are mean ± SEM. Asthma group *n* = 22; control group *n* = 17. Control, ASM cells that had not been subject to co-culture with eosinophils; integrin-blocking, 1-h incubation with Arg-Gly-Asp-Ser (RGDS)/Gly-Arg-Ala-Asp-Ser-Pro (GRADSP) in a 0.125 mg/mL final concentration. Statistical significance, *p* < 0.05.

After ASM cells were incubated with eosinophils from the asthma group and integrins were blocked, ASM cell proliferation reduced to 11.52 ± 2.50%, while eosinophil integrin-blocking in the control group showed that the proliferative effect was reduced to 2.85 ± 0.82% (Figure 6).

## Discussion

In this study we examined ASM remodeling in patients with asthma and found that adhesion of eosinophils from steroid-free asthma patients to ASM cells is important to induce ASM cell proliferation after enhancing ECM protein production, and that this effect can be controlled by the suppression of eosinophil integrins. Eosinophils in asthma patients have significantly increased activity compared with eosinophils in healthy people, showing increased expression of outer-membrane integrins, exhibiting overexpression of TGF-β_1_, having increased adhesive properties, and having stronger effects on inducing gene expression of TGF-β_1_, WNT-5a, and collagen in ASM cells. These effects can be controlled using the small tetrapeptide RGDS, which can non-specifically suppress eosinophil integrins and reduce eosinophil adhesion, thereby influencing the ASM remodeling process.

Airway smooth muscle (ASM) remodeling is associated with varying levels of fibrosis, which is a result of increased deposition and decreased degradation of ECM proteins (Araujo et al., 2008). Both major symptoms of ASM remodeling (increased ASM mass and ECM deposition) are closely related to chronic airway inflammation and many authors correlate asthma severity with eosinophil counts in the sputum or bronchoalveolar lavage fluid (Bousquet et al., 1990; Loutsios et al., 2014).

While it is hard to manage eosinophil migration into the airways, it may be possible to control eosinophil-induced local inflammation. We hypothesized that one possible way to influence the severity of asthma is by blocking eosinophil contact with ASM cells or released ECM proteins.

By measuring eosinophils adhesion we noticed that blocking of eosinophil integrins had similar effect on both, asthmatic and healthy eosinophils groups and the low decrease in eosinophil adhesion can be explained by different types of adhesion properties, for example, cell–cell or cell–ECM protein contact. However less affected eosinophils attachment may not reflect the true integrin blocking effect concerning wide integrins functions.

Integrin adhesion can act as a signal transducer and can control cell processes, such as cell growth, division, survival, cellular differentiation, and apoptosis (Kim et al., 2011; Carbonell et al., 2013). Different types of eosinophil integrins have different roles in the control of cellular processes (Barthel et al., 2008; Johansson, 2014), and blocking RGDS-binding integrins may have a significant effect on ASM cell activity changes.

Increased asthmatic eosinophils adhesion could be explained in part by our determined increased α_4_β_1_ and αMβ_2_ integrins expression. α_4_β_1_ has been studied more extensively than any other eosinophil integrin because of its role in mediating adhesion (Barthel et al., 2006a) of purified blood or airway eosinophils to VCAM-1 which is integrin counter-receptor upregulated in the asthmatic lung. αMβ_2_ integrin is very useful to investigate increased eosinophil adhesion because recognition of αMβ_2_ is influenced greatly by activation with IL-5, which enhances αMβ_2_-mediated static adhesion of blood eosinophils to ICAM-1 or modules 1 or 4 of VCAM-1 (Sano et al., 2005; Barthel et al., 2006b). This is important because there is a significant increase in blood levels of IL-5 when eosinophils are activated *in vivo* after their release from bone marrow (Joseph et al., 2004).

Eosinophils show increased TGF-β_1_ production, but are not the only source of this growth factor in the lungs, there are reports that ASM cells also produce this cytokine (Lee et al., 2006; Januskevicius et al., 2016). How eosinophils induce overexpression of TGF-β_1_ via ASM cells remains unknown. Zagai with colleagues demonstrated that eosinophil cationic protein released by eosinophil can promote TGF-β_1_ expression by human lung fibroblasts after 2–48 h of incubation (Zagai et al., 2007). To examine this possibility, we studied proteins levels of ECP in co-cultures of growth medium containing eosinophils and ASM cells after 24 h. The results are interesting as the highest concentration of ECP protein was found in co-cultures of eosinophils and ASM cells from healthy control participants. This finding may be explained by degranulation of eosinophils isolated from peripheral blood, which has less ECP protein in the granules because. Under asthma conditions, a person has increased CCL type cytokines concentration (Teran, 2000), which gives more degranulation-promoting signals to eosinophils compared with eosinophils from healthy people (Lintomen et al., 2008).

We found that blocking eosinophil adhesion with RGDS peptide reduced eosinophil degranulation, evidenced by the lower levels of ECP protein observed in co-culture growth medium in both the asthma and control groups. It suggests an idea that blocking of integrins can prevent ASM remodeling by reducing eosinophils degranulation instead of acting only as adhesion reducing agent. Moreover, reduced TGF-β_1_ concentration and significant differences between eosinophils that were subject to integrin-blocking and eosinophils that were not subject to integrin-blocking following a 24-h incubation suggest that integrin suppression may be a potential method to control eosinophil-induced TGF-β_1_ expression in ASM cells in asthma.

One of latest findings suggests that there is crosstalk between TGF-β_1_ and non-canonical Wnt signaling and associated ECM protein deposition (Kumawat et al., 2013). Non-canonical Wnt signaling activated via WNT-5a ligand is involved in adult tissue homeostasis (Logan and Nusse, 2004; Clevers, 2006). ASM remodeling is mainly characterized by increased muscle mass because of cell hyperplasia and hypertrophy, but increased production of specific types of ECM proteins is also an important step in ASM abnormalities in asthma, as the ASM bundle thickens and expresses more ECM and contractile proteins compared with healthy people (Woodruff et al., 2004; Léguillette et al., 2009; James et al., 2012). In the current study, we sought to better understand and to control eosinophil-induced ASM remodeling caused by altered ASM cell proliferation and ECM protein production. Eosinophil integrins were found to be essential to induce the observed effects on *WNT-5a* gene expression. After eosinophil integrin-blocking, *WNT-5a* gene expression decreased to levels of no significance. Kumawat et al. (2013) already showed that TGF-β_1_–Wnt signaling are involved in release of fibronectin and collagen regulation. Our results demonstrate ECM protein production by ASM cells after exposure to eosinophils from asthma and control groups *in vitro*. However, Halwani et al. (2013) suggested that ASM cells proliferation increase irrespective of ECM proteins production in asthma and presume that eosinophils enhance ASM proliferation via the release of CysLTs.

TGF-β_1_ can induce the proliferation of structural cells, and the joining of ASM cells with ECM protein can also be recognized as a proliferation signal. Disturbing the stable adhesion of eosinophils to ASM cells and the subsequent ECM protein production cascade through eosinophil integrin-blocking resulted in decreased eosinophil-induced ASM cell proliferation.

From measurements of ASM cell proliferation after exposure to eosinophils, it was apparent that incubation of eosinophils with RGDS peptide reduced the proliferative effect on ASM cells by more than double compared with eosinophils that were not subject to integrin-blocking. This result suggests the non-specific binding of RGDS peptide to eosinophil integrin, which partly reduces eosinophil adhesion, may significantly decrease eosinophil activity by blocking the eosinophil integrin-activated signaling cascade in ASM cells after binding to their receptors.

These findings are in agreement with previous findings by Dekkers et al. (2010) who showed in animal model that RGDS peptide inhibits ASM remodeling while eosinophil number was not affected. These data together with our results about not high impact to eosinophils adhesion suggest that the RGDS peptide interferes with the way in which eosinophils adhere and signal to ASM cells instead of highly affecting stable eosinophils adhesion. Moreover, findings that adhesion of eosinophils are less affected after integrins-blocking with RGDS peptide, but gene expression levels of *WNT-5a*, TGF-β_1_, ECM proteins as well as cell number are more affected, suggest that cell-cell contact is less important in the functional responses in ASM cells, whereas integrin signaling and indirect action of eosinophils by released mediators may be more important.

In our *in vitro* model used eosinophils from peripheral blood, which may result in differences when compared with the *in vivo* situation, where eosinophils that have an effect on ASM cells migrate from the peripheral blood into the airways and are called airway tissue eosinophils (Liu et al., 1998). Despite that, it is suggested that this model is reliable for use in *in vitro* studies because eosinophils from people with asthma are known to be primed or activated *in vivo* and to show enhanced responses to different stimuli *in vitro* (Liu et al., 2003). Increased proportion of eosinophils-ASM cells *in vitro* differs from *in vivo* context and this may give elevated effects for all findings, however during whole incubation period cultured ASM cells had contact only with fixed number of eosinophils while *in vivo* ASM cells have permanent contact with migrating eosinophils. Consequently the study was designed to highlight the differences between effects of asthmatic and healthy eosinophils on ASM cells. Moreover, most of our results were based only on gene expression studies which did not always reflect similar changes at the protein level.

## Conclusions

The findings suggest that ASM remodeling can be controlled by blocking the activity of eosinophil integrins thereby reducing eosinophil adhesion to, and their effects on, ASM cells. Suppression of eosinophil integrins may provide a potential therapeutic tool to reduce eosinophilic inflammation associated airway remodeling in asthma.

## Author contributions

Conceived and designed the experiments: KM, RG, SV, IJ, and AJ. Performed the experiments: AJ, SV, and IJ. Analyzed the experimental data: AJ, RG, and KM. Take care of patients, analyzed clinical data: DH and KM. Contributed reagents/materials/analysis tools: AJ, IJ, and SV. Revised the manuscript for intellectual content: AJ, RG, RS, SV, IJ, AH, DH, and KM. All authors have read and approved the final manuscript.

## Funding

This study was funded by the Research Council of Lithuania (project number: MIP-010/2014).

### Conflict of interest statement

The authors declare that the research was conducted in the absence of any commercial or financial relationships that could be construed as a potential conflict of interest.

## Availability of data and materials section

The datasets supporting the conclusions of this article are included within the article (and its additional files).

## Abbreviations

ASM: airway smooth muscle
ECM: extracellular matrix
TGF-β_1_: transforming growth factor-β_1_
Wnt: wingless/integrase-1 signaling
Wnt-5a: non-canonical Wnt signaling pathway activating ligand 5a
*WNT-5a*: gene coding Wnt-5a ligand
PCR: polymerase chain reaction
EDTA: ethylenediaminetetraacetic acid
PBS: phosphate-buffered saline
EPO: eosinophil peroxidase
ELISA: enzyme-linked immunosorbent assay
EDTA: ethylenediaminetetraacetic acid
DMEM: Dulbecco's modified eagle medium
ICAM-1: intercellular adhesion molecule-1
VCAM-1: vascular cell adhesion molecule-1
FEV_1_: forced expiratory volume in one second
FVC: forced vital capacity
DTT: dithiothreitol.
